# Cytokeratin 5 and cytokeratin 20 inversely correlate with tumour grading in Ta non‐muscle‐invasive bladder cancer

**DOI:** 10.1111/jcmm.16712

**Published:** 2021-06-29

**Authors:** Tim Muilwijk, Murat Akand, Frank Van der Aa, Vincent De Coninck, Marc Claessens, Robert Hente, Markus Eckstein, Yves Allory, Louis Libbrecht, Steven Joniau, Thomas Gevaert

**Affiliations:** ^1^ Department of Urology University Hospitals Leuven Leuven Belgium; ^2^ Organ Systems KU Leuven Leuven Belgium; ^3^ Department of Urology AZ Klina Brasschaat Belgium; ^4^ Department of Pathology University Hospital Erlangen Erlangen Germany; ^5^ Department of Pathology Curie Institute Paris France; ^6^ Department of Pathology AZ Groeninge Kortrijk Belgium; ^7^ Department of Pathology University Hospital Saint‐Luc Brussels Belgium; ^8^ Department of Pathology AZ Klina Brasschaat Belgium

**Keywords:** basal, CK20, CK5, GATA3, immunohistochemistry, luminal, non‐muscle‐invasive bladder cancer

## Abstract

Cytokeratin 5 is a marker of basal molecular subtypes of muscle‐invasive bladder cancer (MIBC), which correlates with worse overall survival compared to luminal subtypes. Our observations have not confirmed CK5 as a marker of high‐grade (HG) disease in Ta non‐muscle‐invasive bladder cancer (NMIBC). Therefore, to understand the basal‐luminal immunohistochemistry profile in Ta NMIBC, we performed immunohistochemistry for CK5, P40, P63 (basal), GATA3 and CK20 (luminal) and studied the correlation with HG and clinical outcome in 109 patients with Ta NMIBC. HG and low‐grade (LG) diseases were scored in each patient. Four different CK5 patterns were evaluated: absent (median 41.3%), normal (72.5%), rising (84.4%) and full thickness (23.9%). The median percentage of GATA3 was 100%. HG disease and CK5 expression and rising CK5 pattern had a significant inverse correlation, whereas HG disease and CK20 expression had a significant positive correlation. We also found a significant inverse correlation between CK5 expression and CK20 expression. Quantitative PCR confirmed that the presence of CK5 correlated with up‐regulation of CK5 RNA. None of the markers could differentiate patients with regard to clinical outcome. Our results suggest a role for CK5 and CK20 in differentiating between LG and HG disease in Ta NMIBC.

## 1 INTRODUCTION

Bladder cancer (BC) is the seventh most common cancer worldwide in men, with a yearly incidence of approximately 549 000 cases.^1^ Three out of four patients present with non‐muscle‐invasive BC (NMIBC), which can be treated non‐invasively by local resection, additional intravesical instillations and stringent follow‐up.^2^ However, NMIBC progresses to muscle‐invasive BC (MIBC) in 31%‐78% patients depending on clinical and pathological risk factors.^3^ Furthermore, 28% of patients with NMIBC, when treated with intravesical BCG instillations, become BCG‐refractory, which significantly impacts their prognosis.^4^ Treatment of BCG‐unresponsive NMIBC and MIBC is aggressive with either surgical resection of the bladder (cystectomy) or trimodal therapy (maximal transurethral resection, chemotherapy and radiotherapy).

Transcriptional analysis of MIBC has revealed several molecular subtypes, with luminal and basal as the most distinct.^5^, ^6^ Interestingly, patients with tumours of the basal subtype have worse overall survival (OS) compared to patients with the luminal subtype. In MIBC, CK5 has been suggested as a surrogate marker for the basal subtype, whereas CK20 and GATA3 have been linked to the luminal subtype.^5^, ^7^, ^8^ Based on these findings, immunohistochemistry (IHC) using CK5 and GATA3 has been used to stratify patients in MIBC between the luminal and basal molecular subtypes.^9^ Basal MIBC is recognized by its CK5+ and GATA3‐ profile, whereas luminal MIBC has a CK5‐ and GATA3+ profile.^9^ Other commonly used basal markers are P63 and its isoform P40 (ΔNp63). P63 and P40 may act as either an oncogene or a tumour suppressor gene.^10^, ^11^ In MIBC, P40 correlates with high‐grade (HG) disease and higher European Organisation for Research and Treatment of Cancer (EORTC) risk score.^12^ Furthermore, patients with loss of P40 have a higher risk of NMIBC relapse and progression.^12^

In contrast to the findings in MIBC, transcriptional analysis of NMIBC Ta and T1 disease identified a basal subtype with improved progression‐free survival (PFS) compared to the luminal subtype.^13^ Interestingly, this basal NMIBC subtype has increased RNA expression of CK5, whereas luminal NMIBC subtypes have higher RNA expression of CK20, a marker of apical umbrella cells.^13^ The luminal marker GATA3 was expressed at even higher levels in the basal subtype in NMIBC than the other subtypes.^13^ Interestingly, sub‐classification of T1 NMIBC using the RNA expression of CK5 and CK20 can be used for the successful stratification of patients, demonstrating worse recurrence‐free survival (RFS) and PFS in patients with high CK20 and low CK5 expression.^14^

Similarly, our routine pathological observations did not confirm the potential of CK5 IHC as a marker of HG Ta NMIBC. Furthermore, we observed significant inter‐tumour and intra‐tumour heterogeneity of CK5 in Ta NMIBC. Given the contradictory findings reported in the RNA and IHC studies between NMIBC and MIBC, we studied the IHC of the basal and luminal markers in a large group of patients with Ta NMIBC for the first time.^15^ To fully capture the heterogeneity of CK5 expression at the IHC level, we applied a novel approach using different CK5 expression patterns and correlated them with the percentages of HG and low‐grade (LG) disease within one patient. Lastly, we aimed to identify correlations between these CK5 IHC patterns and CK5 RNA expression, tumour grading (%), the other basal/luminal IHC markers and RFS.

## 2 MATERIALS AND METHODS

### 2.1 Patients

Patients with Ta NMIBC diagnosed after transurethral resection of the bladder tumour (TURBT) between May 2016 and February 2019 were included in the study cohort. Ethical approval was obtained from the local ethics committee (Ethics Committee AZ Klina no. OG 146; approval no. 086/200/019). Patients with invasive disease (≥T1), clinical node‐positive disease or metastatic disease at the time of TURBT were excluded from the analysis. Histopathology was reassessed in all cases by an expert uro‐pathologist (TG) blinded to clinical outcome. The following parameters were recorded: tumour stage, tumour grade, presence of carcinoma in situ (CIS) and the presence or absence of detrusor smooth muscle. As heterogeneity of tumour grading within one patient is possible, we scored the tumour grade traditionally as either LG or HG according to the WHO 2004 classification, but also scored the percentage of LG and HG disease within each patient. This scoring is a novel method of assessing HG disease burden that can be used to assess the heterogeneity of tumour grading within a patient. To promote reproducibility, grades were scored in increments of 10%.

A retrospective chart review was performed for the following clinico‐pathological data: age, gender, number of lesions, lesion size, primary vs. recurrent NMIBC and prior intravesical treatment. The following survival outcomes were collected: RFS, PFS, cancer‐specific survival (CSS) and OS. All survival end‐points were defined as the time from date of TURBT; for RFS, the end‐point was the date of first recurrence of disease at cystoscopy, for PFS the date of first diagnosis of ≥T2 disease, for CSS the date of death due to BC, and for OS the date of death due to any cause.

### 2.2 Immunohistochemistry

Depending on the extent of the TURBT, 1 or >1 formalin‐fixed paraffin‐embedded (FFPE) blocks were available per patient. In the case of the availability of >1 FFPE block, we selected a representative FFPE block after the presence of a relevant tumour was assessed on all haematoxylin‐and‐eosin‐stained slides. Sections (4 μm) were cut and mounted on poly‐L‐lysine‐coated glass slides. IHC was performed using the automated Benchmark Ultra IHC system (Roche Diagnostics). The automated procedure consisted of blocking endogenous peroxidase activity using 0.3% H_2_O_2_ in methanol, with heat‐induced epitope retrieval (HIER) in Tris‐EDTA buffer (pH 7.8) at 95°C for 44 minutes (standard CC1). Slides were incubated with primary antibodies for 15 minutes, a peroxidase‐labelled polymer for 30 minutes and then a substrate chromogen (mixed DAB Refine) for 10 minutes. Nuclear counterstaining was performed with haematoxylin.

Antibody clones were selected for their epitope selectivity (see Table 1 for details); all have been extensively validated for clinical diagnostic practice (www.nordiqc.com). Prior to enrolment in the study, antibodies were validated on control tissue (tissue with known endogenous expression) for staining specificity and reliability. The primary antibodies used were anti‐CK5, anti‐GATA3, anti‐P40, anti‐P63 and anti‐CK20 (Table 1).

**TABLE 1:** Properties of the antibody clones used

Immunogen	Clone	Manufacturer/Code	Host	Titre	Control
Cytokeratin 5 (CK5) (Immunizing human cytokeratin 5 protein N‐terminal synthetic decapeptide of α‐smooth muscle actin N‐terminal synthetic decapeptide of α‐smooth muscle actin)	D5/16B4	Roche Diagnostics, Belgium 790‐4554	Mouse	Ready to use	Tonsil, prostate
Cytokeratin 20 (CK20) (C‐terminus of the human cytokeratin 20 protein N‐terminal synthetic decapeptide of α‐smooth muscle actin N‐terminal synthetic decapeptide of α‐smooth muscle actin)	SP33	Roche diagnostics, Belgium 790‐4431	Mouse	Ready to use	Colon
GATA3 (Specific immunogen not provided)	L50/823	Roche diagnostics, Belgium 760‐4897	Mouse	Ready to use	Breast, urothelium
P40 (amino acid sequence from amino acid 5 to 17; PVLEPGDKPRKAT)	BC28	Roche diagnostics, Belgium 790‐4950	Mouse	Ready to use	Prostate (basal cells)
P63 (N‐terminus of the human P63 proteinN‐terminal synthetic decapeptide of α‐smooth muscle actin N‐terminal synthetic decapeptide of α‐smooth muscle actin)	4A4	Roche diagnostics, Belgium 790‐4509	Mouse	Ready to use	Prostate (basal cells)

**FIGURE 1:**
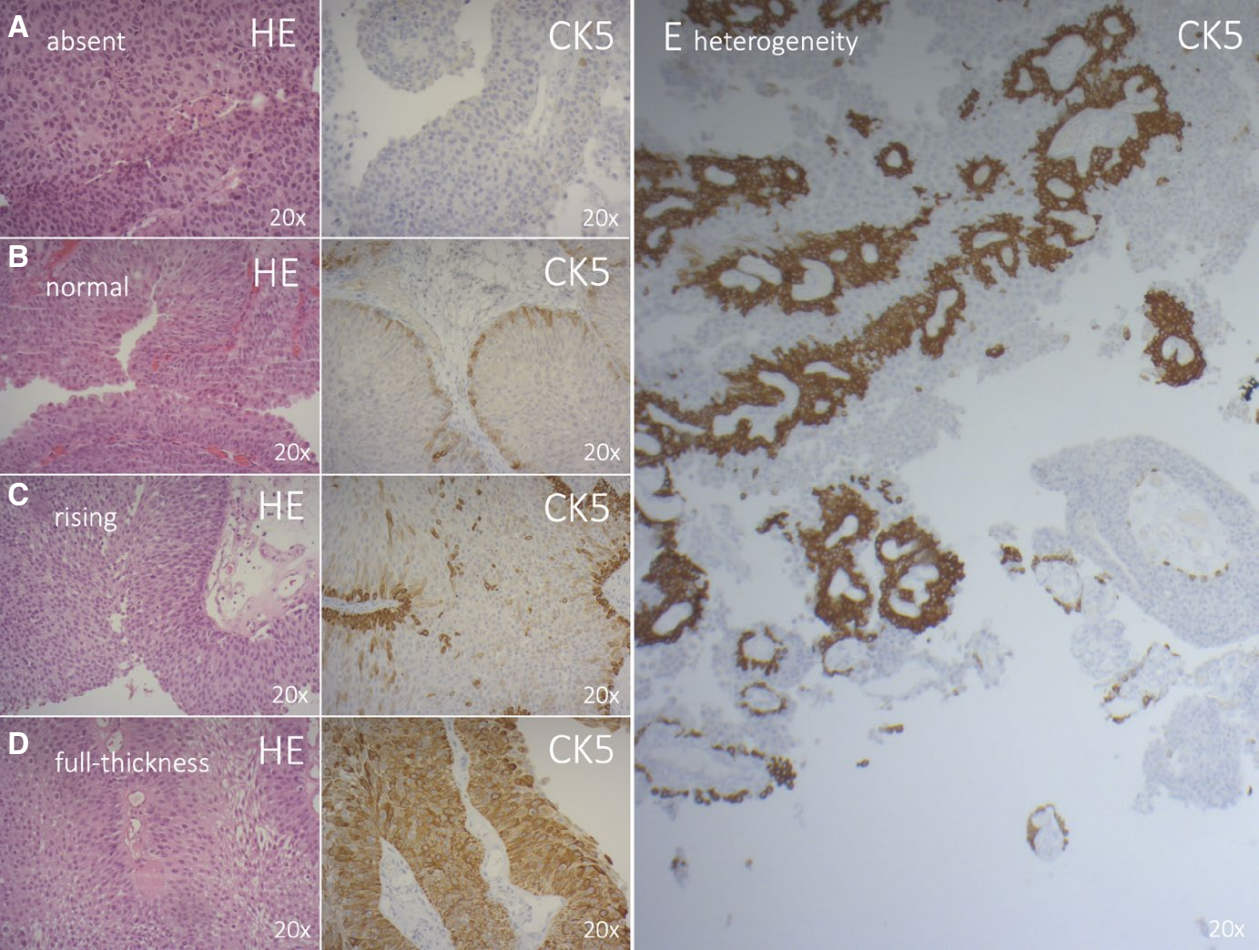
Patterns of CK5 IHC expression: A, absent, B, normal, C, rising and D, full thickness. For each pattern: on the left side haematoxylin and eosin stain and on the right side CK5 stain; on 20x magnification. E: Illustration of heterogeneity of CK5 IHC expression patterns present

**FIGURE 2:**
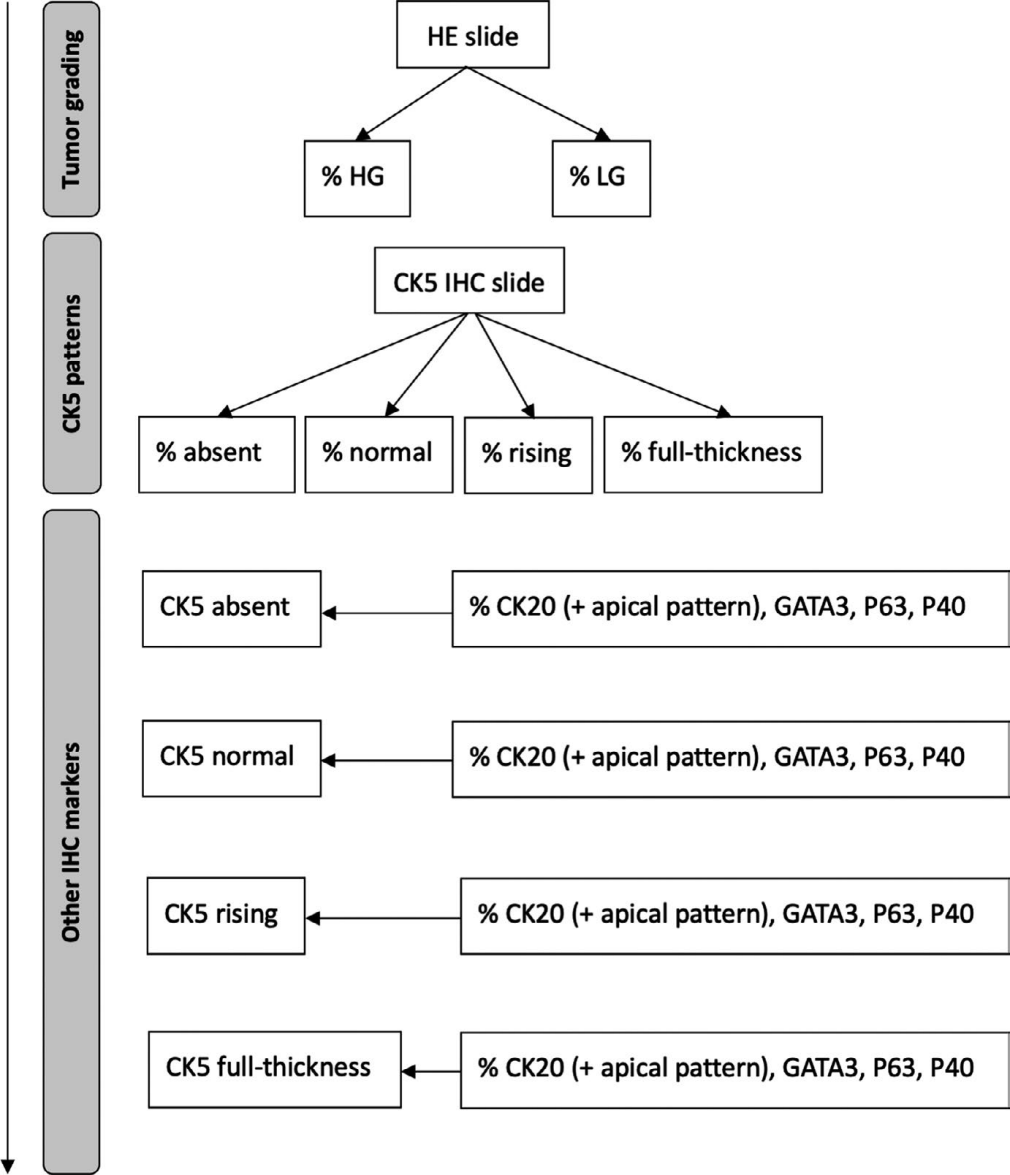
Workflow to assess intra‐tumour heterogeneity by scoring tumour grading, CK5 IHC expression patterns and other IHC markers. Firstly, tumour grading was assessed on an HE slide and scored by % of presence of HG and % of presence of LG disease. Secondly, CK5 patterns were assessed on a CK5 IHC slide by scoring each % of presence of each pattern per patient. Lastly, for each CK5 pattern, we assessed IHC expression of the other luminal and basal markers by scoring % of presence of each IHC marker per CK5 expression pattern

Cytokeratin 5 is a marker of undifferentiated basal cells in the urothelium and has been correlated with the basal molecular subtype in RNA expression studies in both MIBC and NMIBC. Based on the clinical observation by our uro‐pathologists (TG and LL) that CK5 expression can be heterogeneous within each patient, we developed a scoring system for CK5 expression using different patterns to fully capture the heterogeneity of CK5 IHC within each patient. The CK5 expression pattern in IHC could be either ‘absent’ (ie no CK5 expression present), or ‘normal’, ‘rising’ or ‘full‐thickness’ when CK5 expression was present. These four CK5 expression patterns are illustrated in Figure 1. The normal pattern is CK5 in the basal layer, as is expected from a basal marker, whereas the rising pattern comprises CK5 in the basal layer with increasing CK5 expression towards the luminal side. Comparable expression patterns have been described in both NMIBC and MIBC, which strengthens our methodological approach.^16^ To capture heterogeneity, we scored the presence of each pattern as the percentage of each pattern per patient using a visual estimation (Figure 2). In addition, we scored the presence of the other basal (P40, P63) and luminal (GATA3, CK20) markers per CK5 pattern present in each patient (Figure S1). Finally, an additional assessment was made of the apical expression pattern of CK20; in normal urothelium, CK20 is present in umbrella cells only. Based on observational findings, we discriminated three patterns (apical only, apical negative, and apical attenuated), and the percentages of these patterns were scored per CK5 pattern (Figure S2).

Our workflow of assessing heterogeneity in tumour grading, CK5 IHC patterns and other IHC markers is illustrated in Figure 2. IHC staining was evaluated and scored by a specialized uro‐pathologist (TG).

### 2.3 Real‐time quantitative polymerase chain reaction

To correlate the results of CK5 IHC with CK5 RNA expression, we used the sample function in R to randomly select 10 samples with the presence of any CK5 expression based on IHC (combination of ‘normal’, ‘rising’ or ‘full‐thickness’) and 10 samples that had >50% absence of CK5 expression. As all patient samples had some CK5 expression based on IHC, it was impossible to select patients without CK5 expression. Total RNA was extracted from 80‐μm tissue slides using the RecoverAll Total Nucleic Acid Isolation Kit (Life Technologies Corporation) according to the manufacturer's instructions. The RNA integrity number (RIN; 28S/18S) and RNA concentration were assessed using the Experion RNA StdSens (Bio‐Rad) according to the manufacturer's instructions. Only samples with a concentration >50 ng/μL were selected for further downstream experiments.

First‐strand cDNA was synthesized from 500 ng of RNA by reverse transcription using the High Capacity cDNA Reverse Transcription Kit (Applied Biosystems, Thermo‐Fisher) according to the manufacturer's protocol. Real‐time qPCR was performed using the TaqMan^®^ PCR kit (Thermo‐Fisher). TaqMan assay primers and probes (Assays‐on‐Demand Gene Expression Products) were obtained (Applied Biosystems, Thermo‐Fisher). The reaction mixture (25 μL) containing 1 μL of cDNA template, 1 μL of each primer and probe mix and TaqMan Universal PCR master mix (Applied Biosystems, Thermo‐Fisher) was amplified as follows: denaturation at 95°C for 10 minutes and 40 cycles of 95°C for 10 s and 60°C for 20 s. Direct detection of PCR products was monitored by measuring the fluorescence produced by the result of TaqMan probe hydrolysis after every cycle. Data were analysed using the 2–ΔΔCt method for relative quantification compared to GAPDH and S18 as internal controls.

### 2.4 Statistical analysis

Analyses were performed in R (version 3.6.0) using the ‘tidyverse,’ ‘lubridate,’ ‘survival,’ ‘survminer,’ ‘pheatmap,’ ‘Hmisc’ and ‘corrplot’ packages. Summary statistics are presented for continuous variables as median with interquartile range (IQR) and for categorical variables as frequencies and proportions. Spearman correlation coefficients were used to assess the relationship between continuous variables of interest: percentage of CK5 expression patterns, percentage of LG vs. HG and expression of IHC markers. Spearman correlation was used due to the variables of interest being skewed and having outliers. The normality of variables was assessed using the Lilliefors normality test. A heatmap was created using complete‐linkage clustering, which is a method of agglomerative hierarchical clustering. Kaplan‐Meier plots were used to visualize the survival data. Log‐rank tests and Cox proportional hazard models were used to assess correlations between RFS and categorical or numerical variables, respectively.

## 3 RESULTS

### 3.1 Clinical and pathological characteristics

A total of 109 Ta tumours met our inclusion criteria and were included in the analysis. Relevant clinical and pathological characteristics are summarized in Table 2. The median age at the time of TURBT was 76 years (IQR 67‐82). Median follow‐up was 19 months (IQR 15‐24). HG disease was present in 82% (89/109) of patients, which means that 82% would be considered as having high‐risk NMIBC following the EAU guidelines risk stratification.^2^ The median percentage of HG and LG disease was 50% (IQR 20‐70) and 50% (IQR 30‐80), respectively. CIS was present in one patient (1%). A total of 48 patients (44%) had recurrent disease, six (13%) of whom had prior intravesical BCG. Adjuvant intravesical chemotherapy instillations were administered in 42 patients (39%). Adjuvant intravesical BCG instillations were administered in five patients (5%), three (3%) of whom remained recurrence‐free at follow‐up and two (2%) became BCG‐unresponsive and progressed to MIBC. A total of 35 patients (32%) had disease recurrence at follow‐up, four (4%) of whom progressed to MIBC. These four patients had recurrent disease at inclusion, and three of them had BCG‐refractory T1 HG disease at TURBT.

**TABLE 2:** Clinical and pathological characteristics of all patients

	No.	%/[IQR]
Total patients	109	100
Unique patients	95	NA
Median follow‐up (mo)	19	[15‐24]
Age at time of TURBT		
Median	76	[67‐82]
≤65	18	17
>65	91	83
Gender		
Male	89	82
Female	20	18
Tumour stage		
Ta	109	100
Tumour grade (WHO 2004)		
HG presence in patients (n)	89	82
Median % of tumour HG (%)	50	[20‐70]
LG presence in patients (n)	96	88
Median % of tumour LG (%)	50	[30‐80]
Carcinoma in situ		
Present	1	1
Absent	108	99
EAU risk categories		
High	90	83
Intermediate	10	9
Low	8	7
NR	1	1
Detrusor in resection specimen		
Present	78	71
Absent	29	27
NR	2	2
Number of lesions		
≥8	24	22
2‐7	22	20
1	61	56
NR	2	2
Size of lesions (cm)		
≥3	21	19
<3	86	79
NR	2	2
Single post‐operative instillation of CHT		
Yes	75	69
No	32	29
NR	2	2
Chemotherapy maintenance		
Yes	42	38
Median number of courses	7	[6‐9]
No	63	58
NR	4	4
BCG induction		
Yes	5	5
Median number of courses	6	[6‐6]
Followed by BCG maintenance	3	3
Median number of courses	3	[3‐6]
No	99	91
NR	2	2
Primary vs. recurrent disease		
Primary	59	54
Recurrent	48	44
Median time to recurrence (mo)	14	[8‐40]
≤1 recurrence/y	20	42
>1 recurrence/y	28	58
NR	2	2
Prior highest tumour stage (recurrent disease‐only; n = 48)		
Ta	36	75
T1	9	19
Tis	1	2
Tx	1	2
NR	1	2
Prior tumour grade (recurrent disease‐only; n = 48)		
G1	28	58
G2	3	6
G3	12	25
LG	2	4
NR	3	6
Prior intravesical instillations (recurrent disease‐only; n = 48)		
BCG	6	13
Chemotherapy	13	27
None	26	54
NR	3	6
Outcome		
Recurrence of NMIBC	35	32
Progression to MIBC	4	4
Death	4	4
Cancer‐specific death	1	1

Abbreviations: BCG, Bacillus Calmette‐Guerin; CHT, chemotherapy; HG, high grade; IQR, interquartile range; LG, low grade; NR, not reported; TURBT, transurethral resection of bladder tumour; WHO, World Health Organization.

### 3.2 Immunohistochemistry and CK5 expression patterns

All Ta NMIBC samples presented CK5 expression by IHC, but with variable intra‐tumoural heterogeneity. We correlated the percentage of any CK5 IHC, regardless of the pattern, with the percentage of HG disease and identified a moderate inverse correlation (ρ) of −0.31 (*P* = .001). We also correlated each CK5 IHC pattern with the percentage of HG disease and identified an inverse correlation between HG disease and the percentage of rising pattern (ρ = −0.28; *P* = .003).

**FIGURE 3:**
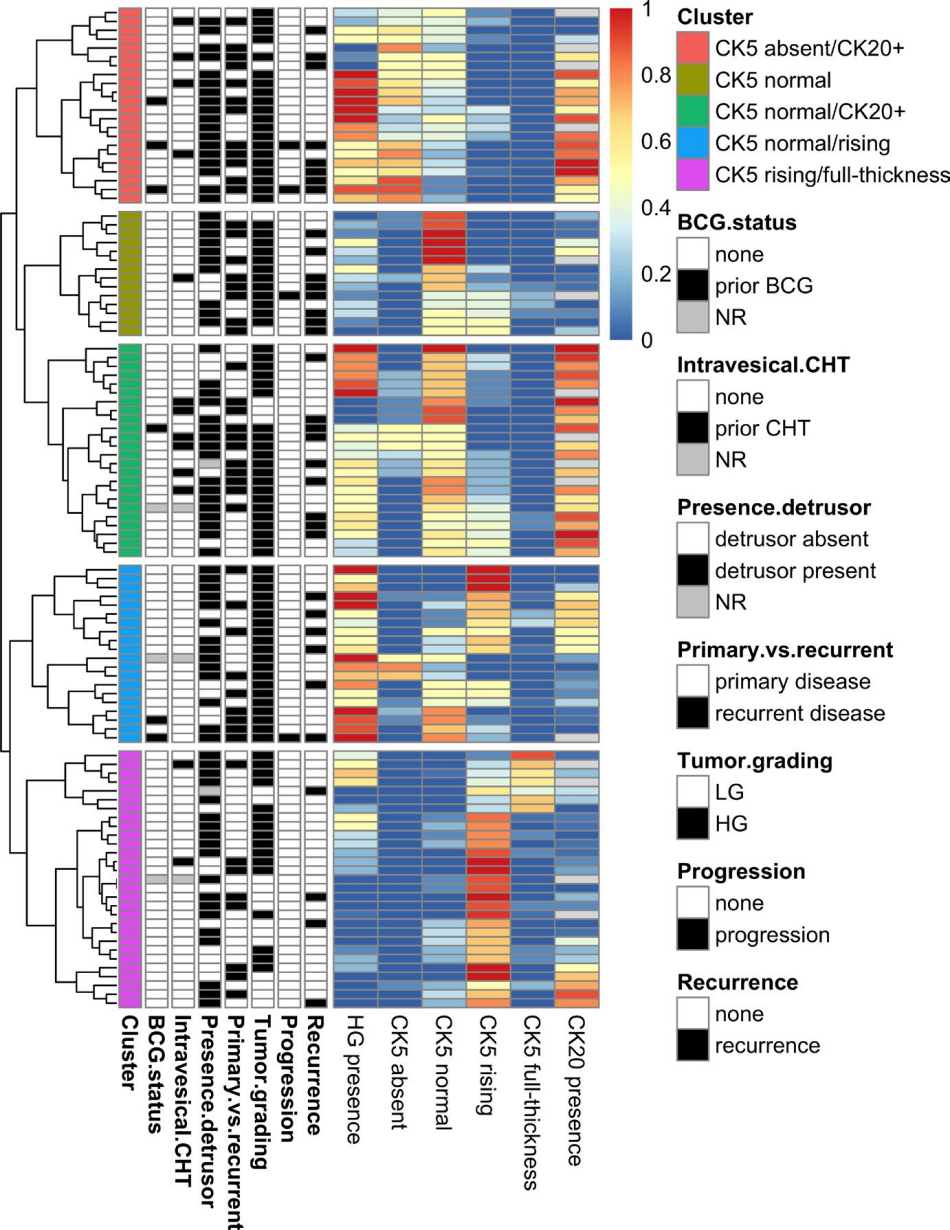
Heatmap using hierarchical clustering for % of presence of HG disease, % of presence of CK5 patterns and % of presence of CK20, we identified five distinct clusters. Clusters from top to below are as follows: CK5 absent/CK20+, CK5 normal, CK5 normal/CK20+, CK5 normal/rising and CK5 rising/full thickness

Using hierarchical clustering for the percentages of HG disease, CK5 patterns and CK20, we identified five distinct clusters (Figure 3): CK5 absent/CK20+, CK5 normal, CK5 normal/CK20+, CK5 normal/rising and CK5 rising/full thickness. Interestingly, patients with a high percentage of HG disease mostly had an absence of CK5 or normal CK5 expression, whereas patients with a low percentage of HG disease had mostly rising and full‐thickness CK5 expression (Figure 3). The full‐thickness CK5 expression pattern almost did not co‐occur with the other CK5 expression patterns, as is visible in the first cluster in Figure 3. More detailed data regarding the Spearman correlation between the percentage of HG and CK5 patterns are given in Table S1.

Next, we looked for correlations between the CK5 expression patterns and the other IHC markers (CK20, GATA3, P40, P63; Table 3). The luminal marker GATA3 and basal markers P40 and P63 were diffused and uniformly expressed in Ta NMIBC (only umbrella cells were consistently P40‐ and P63‐), whereas the expression of luminal marker CK20 was highly variable (median 50%; IQR 5‐75; Table 4). No significant correlations were found between the expression of any of these markers by IHC. As GATA3, P40 and P63 presented uniform expression in Ta NMIBC, no difference in correlation was observed between their expression patterns and the different CK5 patterns. For CK20, we found a moderate inverse correlation (ρ = −0.28; *P* = .01) with the percentage of any CK5 expression (normal, rising, and full‐thickness combined) and the percentage of normal CK5 (ρ = 0.25; *P* = .02; Figure 3). Furthermore, we found a positive correlation between the percentage of CK20 and percentage of HG disease (ρ = 0.21; *P* = .04). The findings regarding CK5, CK20 and tumour grading are further illustrated in Figure S3.

**TABLE 3:** Median percentage of presence of expression of immunohistochemical markers (GATA3, P40, P63 and CK20) per CK5 pattern (absent, normal, rising and full thickness)

CK5 pattern	Median %	IQR	Missing	Completion %
Absent n = 45 (41%)				
GATA3	100	100‐100	2	96
P40	90	80‐90	24	47
P63	90	90‐95	38	16
CK20	78	25‐90	7	84
umbrella +ve	0	0‐0		
umbrella ‐ve	80	50‐100		
accentuated	5	0‐45		
Normal n = 92 (84%)				
GATA3	100	100‐100	5	95
P40	90	88‐95	52	44
P63	90	90‐95	80	13
CK20	50	10‐80	12	87
umbrella +ve	0	0‐10		
umbrella ‐ve	80	50‐100		
accentuated	3	0‐40		
Rising n = 79 (72%)				
GATA3	100	100‐100	4	95
P40	90	90‐95	44	44
P63	90	90‐95	68	14
CK20	25	5‐75	11	86
umbrella +ve	0	0‐20		
umbrella ‐ve	70	43‐100		
accentuated	0	0‐43		
Full‐thickness n = 26 (24%)				
GATA3	100	100‐100	3	89
P40	93	90‐95	16	39
P63	90	90‐93	22	15
CK20	15	5‐29	4	85
umbrella +ve	10	0‐30		
umbrella ‐ve	60	30‐98		
accentuated	5	0‐44		

Note

Percentages of CK20 patterns (umbrella cells positive, umbrella cells negative and accentuated) within the present CK20 as is illustrated in Figure S2.

Abbreviations: +ve, positive; IQR, interquartile range; n, number; NA, not available; ‐ve, negative.

**TABLE 4:** Presence and median percentage of presence of immunohistochemical markers and numbers regarding completion of percentage

IHC marker	Presence (%)	Presence (n)	Median %	IQR	Missing	Completion %
CK5	100%	109	100	70‐100	0	100
GATA3	100%	103	100	100‐100	6	95
P40	100%	47	90	90‐95	62	43
P63	88%	15	90	90‐95	92	16
CK20	87%	82	50	5‐75	15	86
umbrella +ve			0	0‐18		
umbrella ‐ve			77	40‐100		
accentuated			2	0‐40		

Abbreviations: IHC, immunohistochemistry; IQR, interquartile range.

We also assessed the differences in IHC and CK5 expression patterns between patients with primary (n = 59) and recurrent disease (n = 48; Table S2). Any CK5 expression differed significantly (*P* = .03), as well as CK5 full‐thickness expression (*P* = .02). We performed a similar analysis based on EAU risk categories for patients with low‐risk (n = 8), intermediate‐risk (n = 10) or high‐risk disease (n = 90; Table S3). We found no significant differences between EAU risk categories in IHC and CK5 expression patterns. The CK5 full‐thickness pattern was present in 8% of cases (n = 4) with recurrent disease. The other IHC markers (GATA3, P40, P63 and CK20) and CK5 patterns did not differ significantly between patients with primary and recurrent disease.

We examined the correlation between the relative CK5 RNA expression levels and CK5 IHC patterns and found positive correlations between CK5 RNA expression and the presence of any CK5 in IHC (ρ = 0.79; *P* = .0008), and between CK5 RNA expression and the presence of the rising CK5 pattern (ρ = 0.74; *P* = .002; Figure S4).

### 3.3 Survival analysis

**FIGURE 4:**
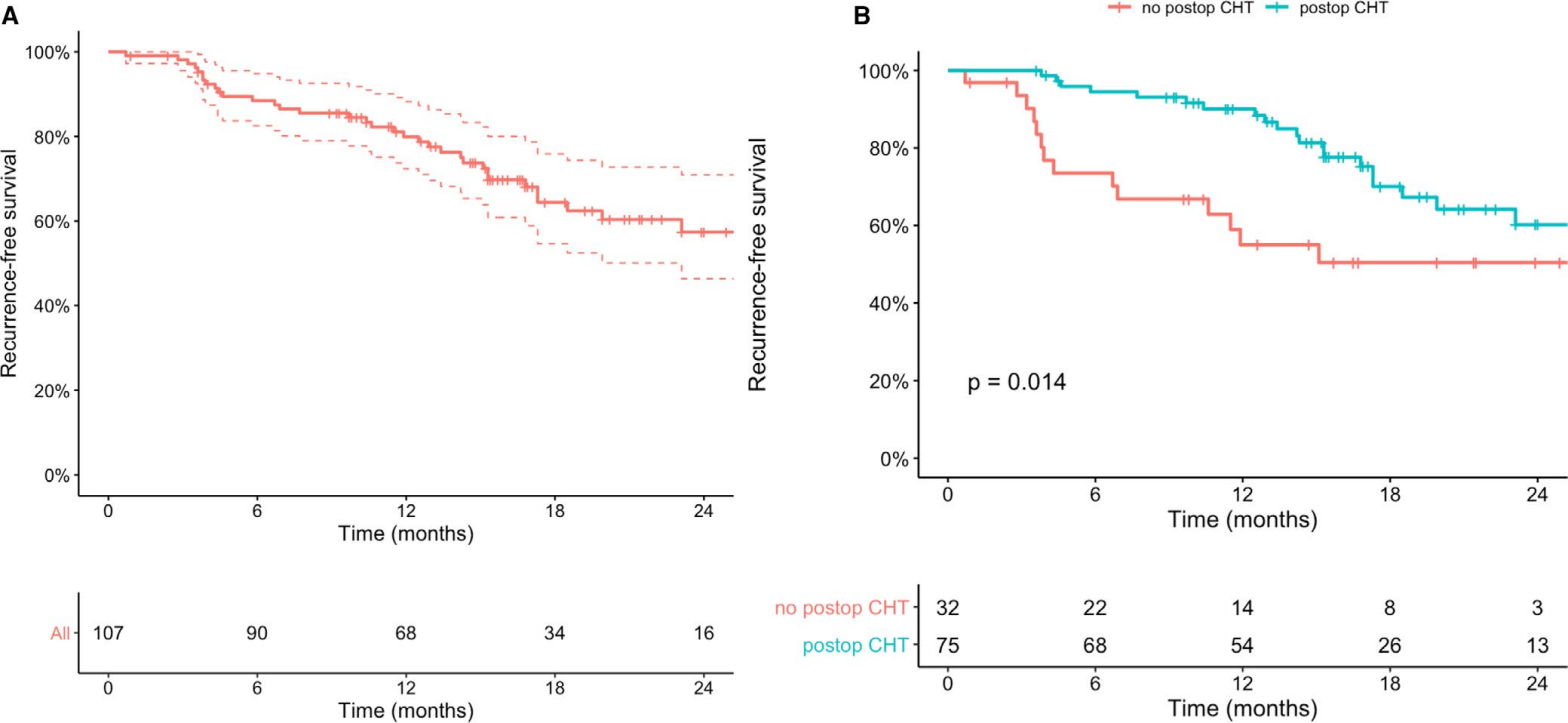
A, Kaplan‐Meier plot of recurrence‐free survival (RFS) of all patients with follow‐up data (n = 107). RFS at 1 year and 2 years was 80% (95% CI: 72‐88) and 57% (95% CI: 46‐71), respectively. Median RFS was not reached (95% CI: 23.1‐NR). B, Kaplan‐Meier plot of RFS of all patients with follow‐up data stratified by administration of a single post‐operative instillation of intravesical chemotherapy (n = 107). RFS in the not administered group at 1 year and 2 years was 55% (95%CI: 39‐77) and 50% (95%CI: 35‐73); and in the administered group 90% (95%CI: 83‐97) and 60% (95%CI: 47‐77), respectively. Median RFS was not reached in both groups

Recurrence‐free survival at 1 and 2 years (Figure 4A) was 80% (95% CI 72‐88) and 57% (95% CI 46‐71), respectively, with a median follow‐up of 19 months (IQR 15‐24). Follow‐up data were missing for two patients. The median RFS was not reached (95% CI 23.1‐NR). Only four patients progressed to MIBC disease, with PFS at 1 and 2 years of 98% (95% CI 96‐100) and 95% (95% CI 90‐100), respectively. CSS at 1 and 2 years was 99% (95% CI 97‐100), and OS at 1 and 2‐years was 96% (95% CI 93‐100). We found no significant effect of any of the CK5 expression patterns or CK5 RNA expression on RFS in univariable or multivariable Cox proportional hazard analyses (Table S4). All clinical, pathological and IHC expression data were checked to identify any prognostic variable for RFS, PFS, CSS and OS in log‐rank (for categorical variables) and Cox proportional hazard analyses (for numerical variables). The five distinct clusters of our heatmap (Figure 3) were not prognostic for RFS (Figure S5), PFS, CSS or OS. The only significant prognostic variable was the post‐operative administration of a single instillation of intravesical chemotherapy, which increased the RFS of patients (hazard ratio: 0.43; 95% CI 0.22‐0.86; *P* = .01; C‐index: 0.63; Figure 4B). We identified no additional prognostic variables when these analyses were performed separately for patients with primary (n = 59) or recurrent disease (n = 48).

## 4 DISCUSSION

Here, we describe the expression patterns of luminal (GATA3, CK20) and basal (CK5, P40, P63) IHC markers in a group of patients with Ta NMIBC. To fully assess the heterogeneity of Ta NMIBC, we used a granular methodology in which we scored the tumour grade as the percentage of HG and LG disease in each patient and addressed four specific patterns of CK5 expression in IHC. The study set‐up is unique because we used a homogenous Ta NMIBC cohort, compared to the often‐used mixed Ta/T1 NMIBC cohorts, and we report four different types of CK5 expression patterns for the first time.

Our findings are novel and interesting for several reasons. First, expression of the basal IHC marker CK5 was present in all patients, with remarkable heterogeneity in the CK5 expression patterns within individual patients. Second, we observed a significant inverse correlation between the percentage of HG disease and expression of CK5. In addition, we confirmed that CK5 protein expression in IHC correlated with CK5 RNA expression. Third, we observed a significant positive correlation between the percentage of HG disease and expression of the luminal IHC marker CK20. We also observed that the luminal marker GATA3 was omnipresent in Ta disease and did not correlate with any specific tumour feature. Fourth, CK5 inversely correlated with CK20. Finally, we found no significant impact of any of the IHC variables on RFS.

In recent years, research on the molecule profile of BC has expanded quickly, with various genomic studies being published. Most of these studies have focused on MIBC, and comprehensive classification schemes have been proposed by different groups.^6^, ^7^, ^8^, ^17^, ^18^, ^19^, ^20^, ^21^, ^22^ Recently, a consensus paper proposed a set of six molecular classes, with the basal/squamous (Ba/Sq) group as the most prominent subgroup (35%).^6^ Although less molecular data are available for NMIBC, three molecular subgroups have been defined by several groups. Hedegaard et al^13^ reported three major tumour classes with basal‐ and luminal‐like characteristics and different clinical outcomes in Ta and T1 NMIBC.^22^ To the best of our knowledge, profiling studies with a unique focus on Ta NMIBC have not been published previously.

Several groups have tried to characterize these molecular subgroups by IHC, and GATA3 and CK5 have emerged as the most frequently used luminal and basal marker, respectively.^9^, ^23^ Our data suggest different biology underlying CK5/CK20 expression in Ta NMIBC and MIBC.^6^ Loss of CK5 and gain of CK20 at the protein level in Ta NMIBC can be hypothesized to mark an evolution to a more aggressive Ta tumour type, whereas the opposite has been reported in MIBC.^6^, ^7^ In MIBC, CK5 expression is a marker of basal differentiation and CK20 of luminal differentiation, which is not the case in Ta NMIBC. The diffuse expression of the basal cell markers P40 and P63 in Ta NMIBC, regardless of the CK5 pattern, supports this thesis. As mentioned above, the role of CK5 in T1 NMBIC is not well elucidated in the literature but, based on current knowledge, we can hypothesize a dichotomous role. In some T1 subgroups, low CK5 could correlate with high‐risk biology, whereas in other T1 subgroups there may be a correlation between high CK5 and high‐risk biology.^22^ This may reflect the existence of two different pathways of CK5 expression in T1 NMIBC. Differences in the molecular biology of NMIBC and MIBC have been reported by others, demonstrating similar gene expression subtypes between NMIBC and MIBC but opposite prognostic results.^13^ Sjödahl et al^15^ reported inconsistencies between mRNA expression and tumour‐cell phenotypes in MIBC but not NMIBC. We observed a concordance between CK5 protein and mRNA expression in Ta NMIBC, which is in line with other reports.^15^, ^23^ Most of the molecular classification studies on BC have used gene expression methods, but the discordant correlations between mRNA and protein levels in MIBC versus NMIBC show that gene expression data should always be validated at the protein level. As suggested by others, a bi‐nominal classification system consisting of both tumour cell phenotype and gene expression clusters would be more appropriate.^15^

Studies on the prognostic value of CK5 expression in NMIBC are rather diffuse. Our study could not link any of the CK5 patterns to recurrence, which is in line with the findings of Lobo et al^23^ Patschan et al^22^ identified a subgroup of T1 NMIBC patients with high expression of CK5 in IHC and poor PFS, but also reported a subgroup with little or no detectable CK5 and poor PFS. In contrast to these findings, Breyer et al^14^ found that the combination of high KRT20 and low KRT5 expression is a significant predictor of worse RFS and PFS in patients with T1 NMIBC. Two separate studies on non‐muscle‐invasive upper urinary tract carcinoma (UTUC) found that CK5 negativity is an independent prognostic factor for shorter PFS and CSS.^24^, ^25^ For both PFS and OS, it was not possible to stratify patients in the present study due to the low number of events, possibly due to the lower risk of progression of Ta disease compared to T1 disease and by the limited follow‐up of 19 months.

This study has several limitations. First, clinical data were retrieved retrospectively, which could have caused a bias in data collection. Second, our cohort consisted of a mix of patients with primary and recurrent disease with HG disease present in 82% of patients. Third, follow‐up was limited to a median of 19 months, and we had relatively few patients progress to MIBC with subsequent cancer‐specific death. Finally, we validated CK5 expression with qPCR in only 14 of the 20 patients, based on a sufficient RNA concentration for further downstream analyses, and we did not perform a standard curve of RNA dilutions due to the limited availability of RNA.

The inverse correlation between CK5 and HG disease, the positive correlation between CK20 and HG disease, the inverse correlation between CK5 and CK20 and the absent correlation between CK5 and GATA3 in our Ta NMIBC cohort support the presence of different molecular biology between NMIBC and MIBC. Our study set‐up is unique in that we used a homogenous Ta NMIBC cohort compared to the often‐used Ta/T1 NMIBC cohorts. The present findings suggest different applicability and relevance of the GATA3/CK5/CK20 panel for the classification and grading of NMIBC compared to MIBC. The added value of these markers to clinico‐pathological grading has to be proven in larger prospectively designed trials with longer term data on clinical outcome. If validated, CK5 and CK20 may serve as additional markers for differentiating between HG and LG Ta NMIBC disease.

## CONFLICT OF INTEREST

The authors declare that they have no conflicts of interest related to this manuscript.

## AUTHOR CONTRIBUTIONS

**Tim Muilwijk:** Formal analysis (equal); Funding acquisition (equal); Investigation (equal); Methodology (equal); Visualization (lead); Writing‐original draft (lead); Writing‐review & editing (equal). **Murat Akand:** Writing‐original draft (supporting); Writing‐review & editing (equal). **Frank Van Der Aa:** Methodology (supporting); Supervision (supporting); Writing‐review & editing (supporting). **Vincent De Coninck:** Investigation (equal); Writing‐review & editing (supporting). **Marc Claessens:** Investigation (equal); Writing‐review & editing (supporting). **Robert Hente:** Investigation (equal); Writing‐review & editing (supporting). **Markus Eckstein:** Writing‐review & editing (supporting). **Yves Allory:** Writing‐review & editing (supporting). **Louis Libbrecht:** Conceptualization (lead); Methodology (lead); Writing‐review & editing (supporting). **Steven Joniau:** Methodology (equal); Writing‐original draft (equal); Writing‐review & editing (equal). **Thomas Gevaert:** Conceptualization (lead); Investigation (equal); Methodology (lead); Supervision (lead); Writing‐original draft (supporting); Writing‐review & editing (equal).

## Supporting information

Fig S1

Fig S2

Fig S3

Fig S4

Fig S5

Table S1‐S4

Supplementary Material

## Data Availability

Data available upon request due to privacy/ethical restrictions.
