# Supplementary figures and images for: Cytokeratin 5 and cytokeratin 20 inversely correlate with tumour grading in Ta non‐muscle‐invasive bladder cancer
Source: J Cell Mol Med. 2021 Jun 29;25(16):7890–900. doi: 10.1111/jcmm.16712 (PMC8358875; doi:10.1111/jcmm.16712)

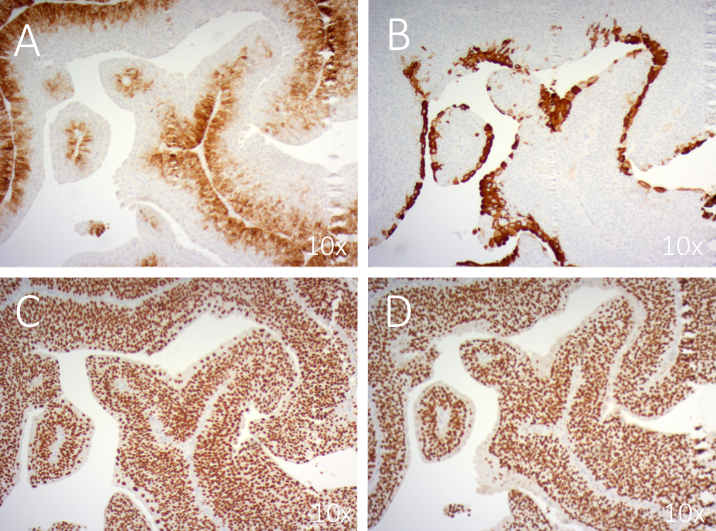

Supplement: Supplementary file 1 — Fig S1 [file JCMM-25-7890-s006.png]

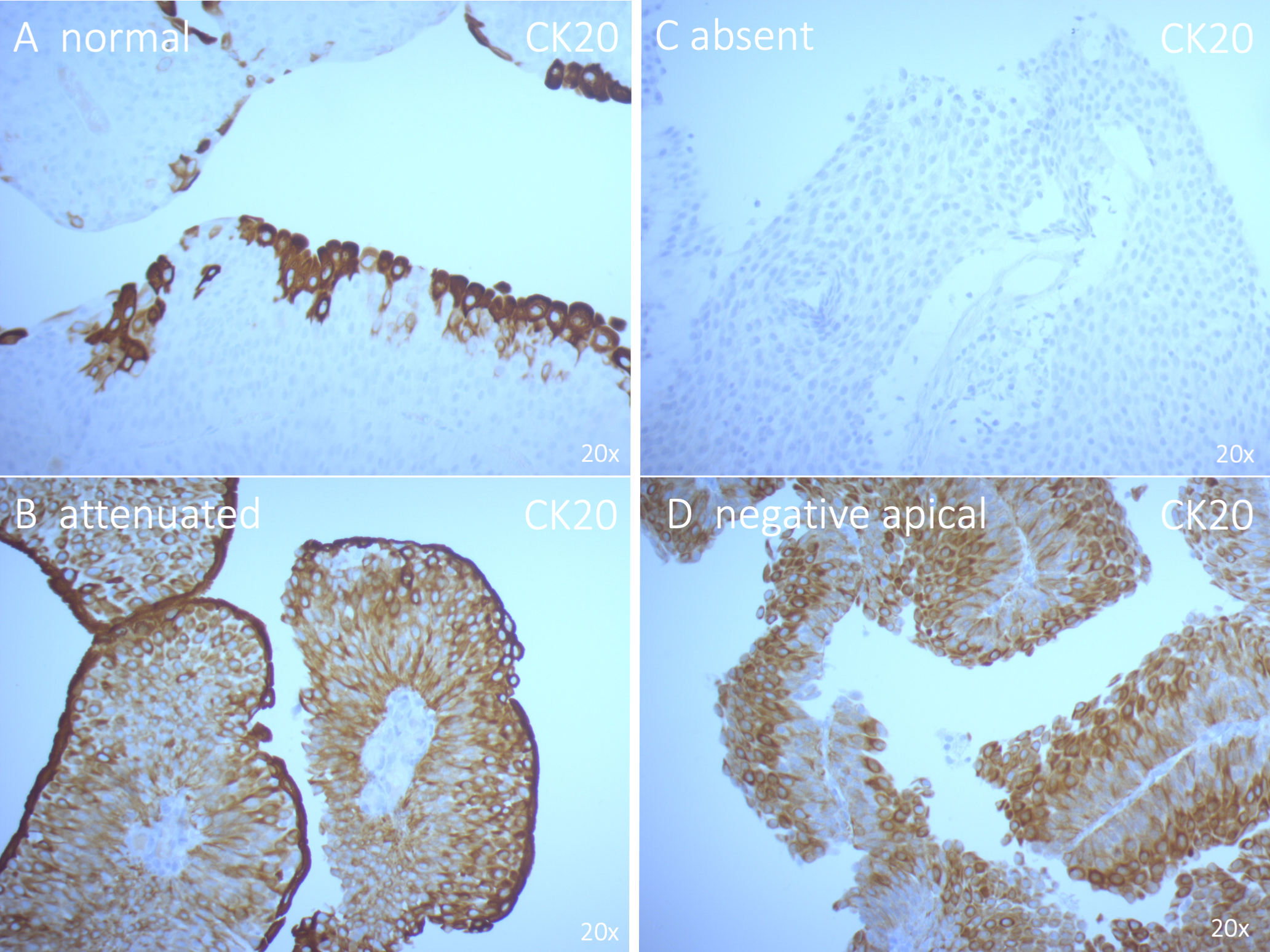

Supplement: Supplementary file 2 — Fig S2 [file JCMM-25-7890-s002.png]

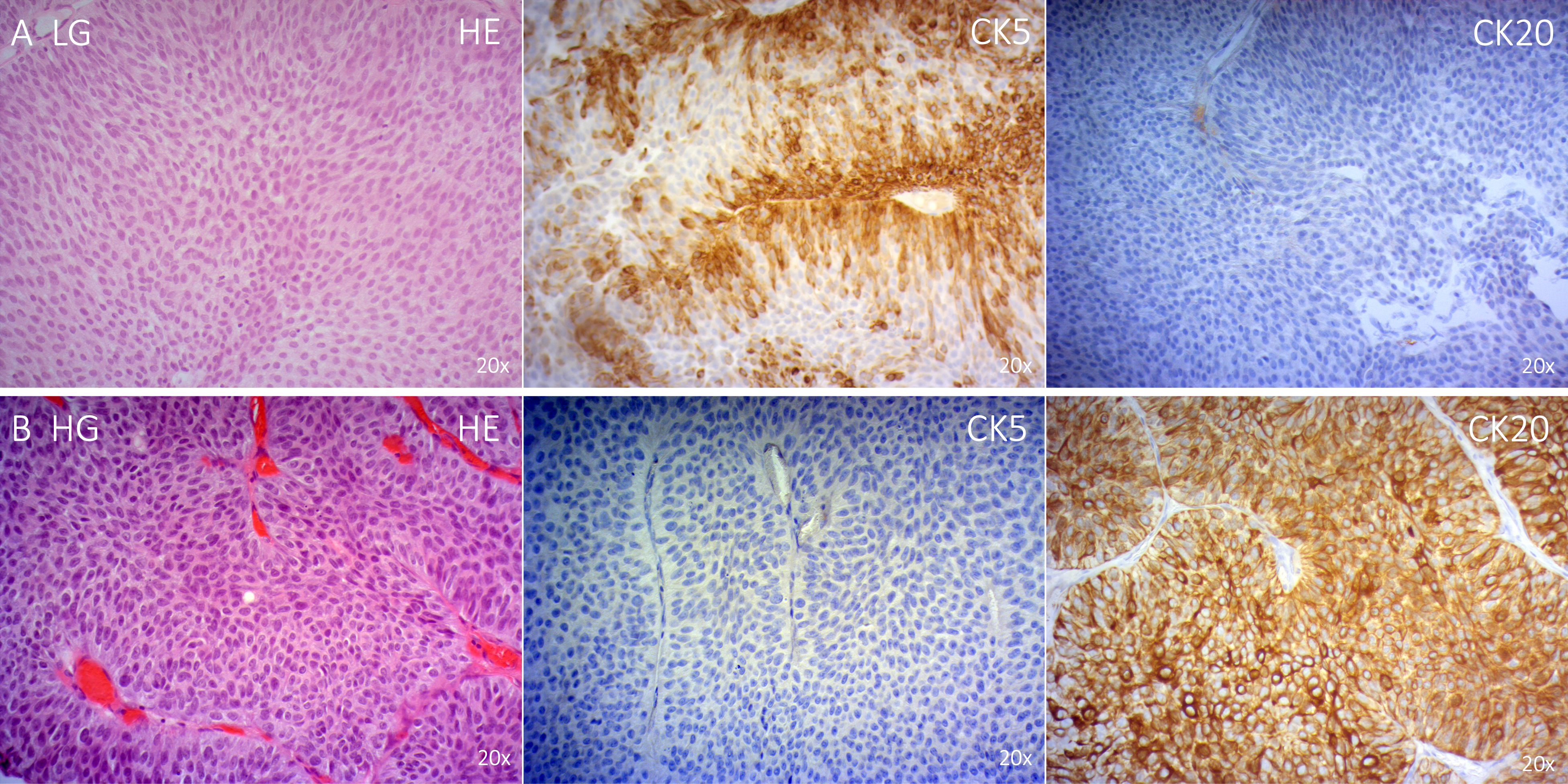

Supplement: Supplementary file 3 — Fig S3 [file JCMM-25-7890-s005.png]

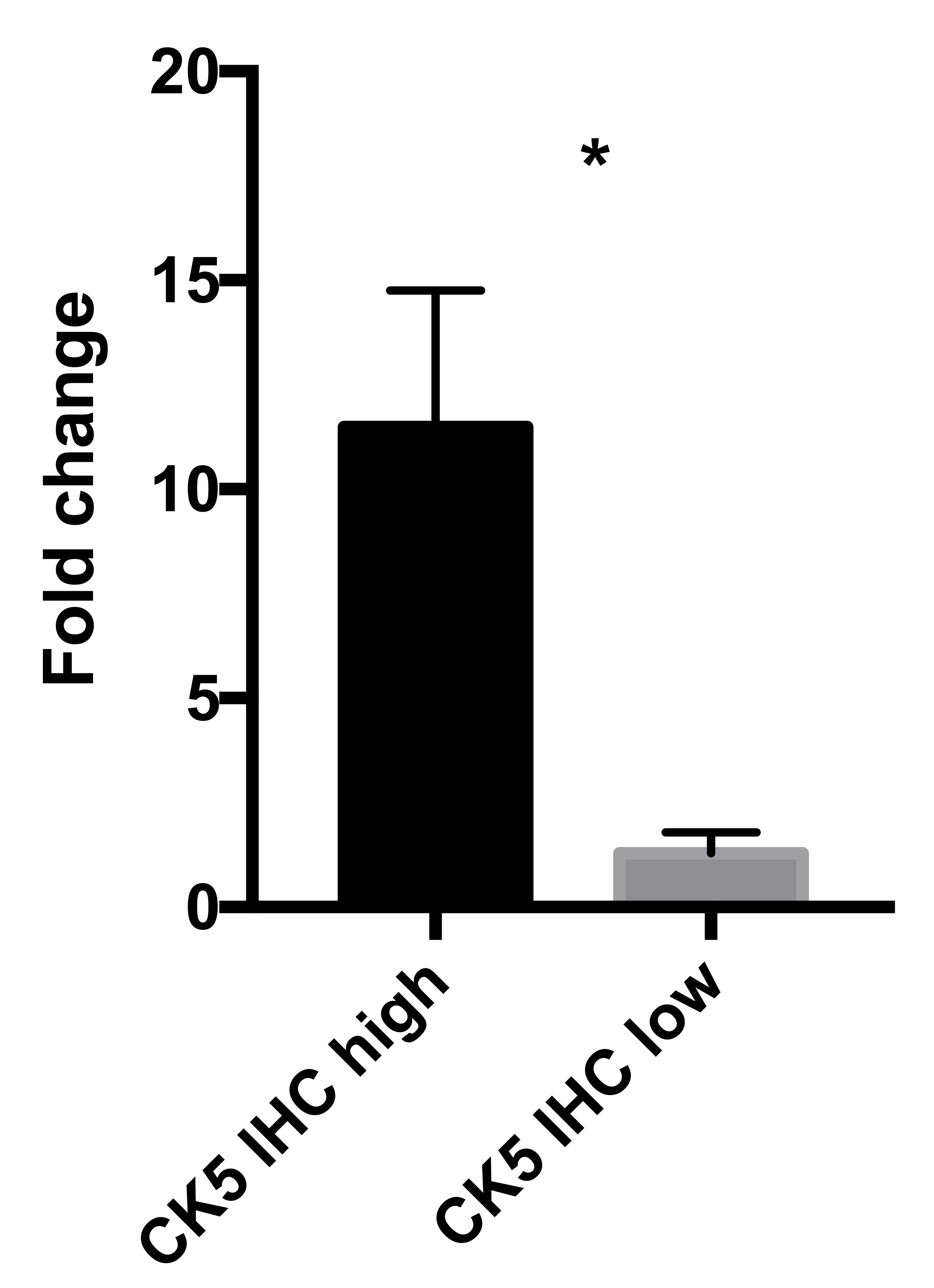

Supplement: Supplementary file 4 — Fig S4 [file JCMM-25-7890-s007.png]

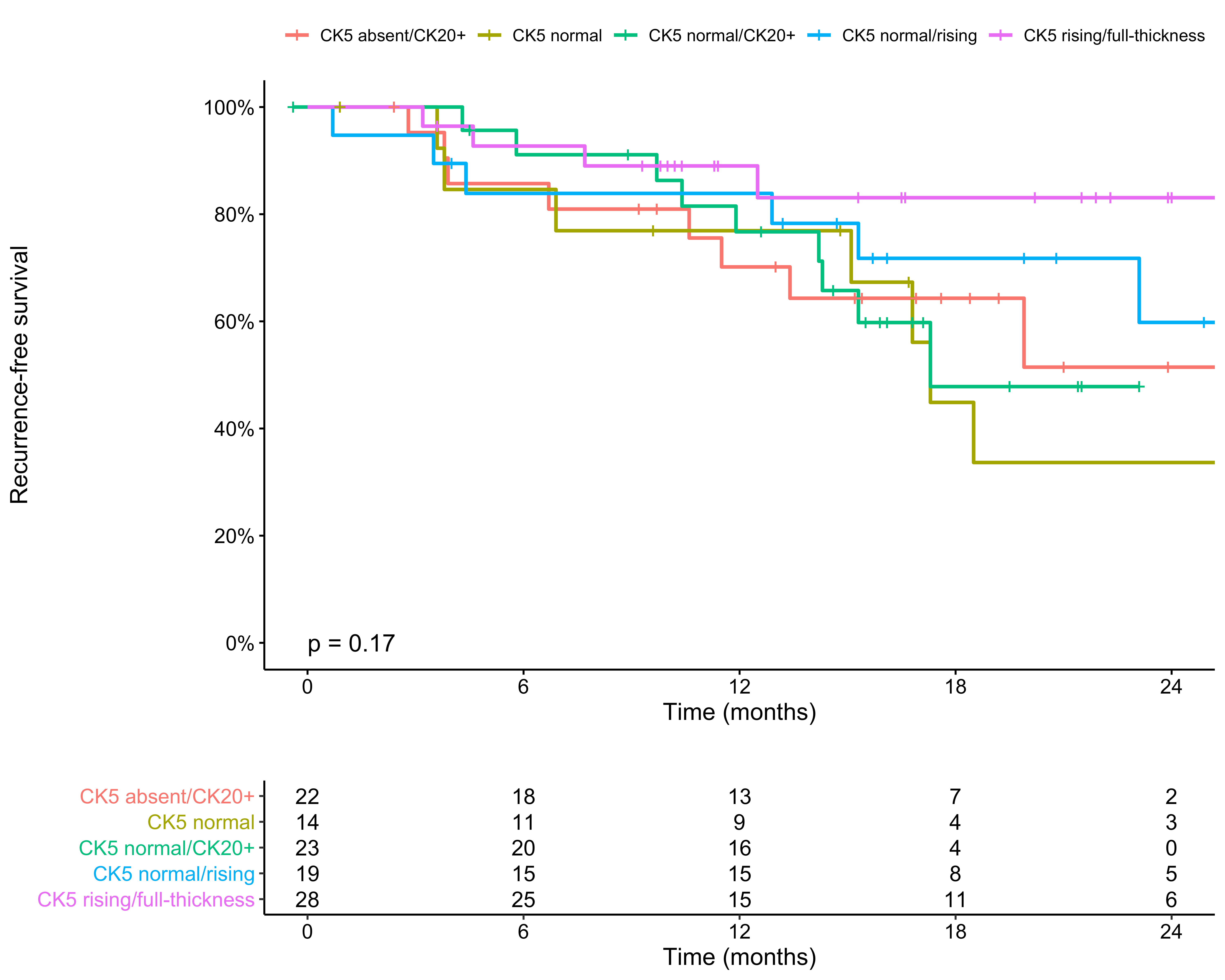

Supplement: Supplementary file 5 — Fig S5 [file JCMM-25-7890-s004.png]
