# Supplementary material for: Cytokeratin 5 and cytokeratin 20 inversely correlate with tumour grading in Ta non‐muscle‐invasive bladder cancer
Source: J Cell Mol Med. 2021 Jun 29;25(16):7890–900. doi: 10.1111/jcmm.16712 (PMC8358875; doi:10.1111/jcmm.16712)
Supplement: Supplementary file 6 — Table S1‐S4 [file JCMM-25-7890-s003.docx]

**Supplementary Table 1:** Spearman correlation index (ρ) between % of HG disease and % of CK5 and CK5 immunohistochemical expression patterns.

| **Correlation with % HG disease** | **ρ** | **p-value** | |
| --- | --- | --- | --- |
| Absent CK5 | 0.31 | 0.001 |  |
| Normal CK5 pattern | 0.17 | 0.07 |  |
| Rising CK5 pattern | -0.28 | 0.003 |  |
| Full-thickness CK5 pattern | -0.18 | 0.06 |  |
| Any CK5 (combined) | -0.31 | 0.001 |  |

Abbreviations: HG: high grade; ρ: Spearman correlation index; p-value: probability value.

**Supplementary Table 2:** Comparison between primary vs. recurrent disease for % of tumor grading, CK5 patterns, and other IHC markers.

|  | **Primary disease** (n=59) | | **Recurrent disease** (n=48) | |  |
| --- | --- | --- | --- | --- | --- |
|  | *median %* | *IQR* | *median %* | *IQR* | *p-value* |
| HG disease | 50 | 25-70 | 50 | 10-70 | 0.7 |
| Any CK5 | 100 | 85-100 | 95 | 50-100 | 0.03 |
| CK5 absent | 0 | 0-15 | 5 | 0-50 | 0.03 |
| CK5 normal | 35 | 10-50 | 50 | 28-70 | 0.1 |
| CK5 rising | 40 | 10-70 | 20 | 0-50 | 0.06 |
| CK5 full-thickness | 0 | 0-10 | 0 | 0-0 | 0.02 |
| GATA3 | 100 | 100-100 | 100 | 100-100 | 0.07 |
| P40 | 90 | 90-95 | 90 | 90-95 | 0.2 |
| P63 | 90 | 90-90 | 90 | 85-95 | 0.3 |
| CK20 | 40 | 5-75 | 55 | 7-75 | 0.9 |

Abbreviations: IHC: immunohistochemistry; IQR: interquartile range; p-value: probability value.

**Supplementary Table 3:** Comparison between European Association of Urology (EAU) risk categories for CK5 patterns, and other IHC markers.

| **EAU risk category** | **High** (n=90) | | **Intermediate** (n=10) | | **Low** (n=8) | |  |
| --- | --- | --- | --- | --- | --- | --- | --- |
|  | *median %* | *IQR* | *median %* | *IQR* | *median %* | *IQR* | *p-value* |
| Any CK5 | 100 | 60-100 | 100 | 90-100 | 100 | 98-100 | 0.2 |
| CK5 absent | 0 | 0-40 | 0 | 0-10 | 0 | 0-2 | 0.2 |
| CK5 normal | 43 | 20-60 | 25 | 0-73 | 23 | 18-30 | 0.4 |
| CK5 rising | 28 | 0-50 | 55 | 3-85 | 70 | 45-76 | 0.06 |
| CK5 full-thickness | 0 | 0-0 | 0 | 0-0 | 0 | 0-5 | 0.7 |
| GATA3 | 100 | 100-100 | 100 | 100-100 | 100 | 100-100 | 0.7 |
| P40 | 90 | 90-95 | 95 | 95-95 | 93 | 91-94 | 0.6 |
| P63 | 90 | 90-95 | 48 | 24-71 | NA | NA-NA | 0.1 |
| CK20 | 50 | 5-75 | 70 | 25-80 | 22 | 4-49 | 0.4 |

Abbreviations: EAU: European Association of Urology; IHC: immunohistochemistry; IQR: interquartile range; p-value: probability value.

**Supplementary Table 4:** Multivariable cox proportional hazards model of CK5 immunohistochemical expression patterns for recurrence-free survival (p = 0.4; C-index = 0.55)

|  | **HR** | **95%CI** | **p-value** |  |
| --- | --- | --- | --- | --- |
| Normal CK5 pattern | 1.50 | 0.32-7.15 | 0.61 | |
| Rising CK5 pattern  Full-thickness CK5 pattern | 0.75  0.28 | 0.20-2.80  0.01-5.42 | 0.67  0.40 | |

Abbreviations: HR: hazard ratio; 95%CI: 95% confidence interval; p-value: probability value.
