# Supplementary material for: Cytokeratin 5 and cytokeratin 20 inversely correlate with tumour grading in Ta non‐muscle‐invasive bladder cancer
Source: J Cell Mol Med. 2021 Jun 29;25(16):7890–900. doi: 10.1111/jcmm.16712 (PMC8358875; doi:10.1111/jcmm.16712)
Supplement: Supplementary file 7 — Supplementary Material [file JCMM-25-7890-s001.docx]

**Supplementary Figure 1:** Patterns of IHC expression of basal and luminal markers: A: CK5, B: CK20, C: GATA3, and D: P40; on 10x magnification. Expression pattern of P63 was similar to that of P40. Similar tissue areas are shown.

**Supplementary Figure 2:** Patterns of IHC expression of CK20: A: apical only, B: apical attenuated, C: absent, and D: apical negative; on 20x magnification. Patterns A and B both express apical CK20 staining, whilst patterns C and D lack apical CK20 staining.

**Supplementary Figure 3:** Illustrative figure showing inverse correlation between tumour grading on HE, % of CK5 on IHC, % of CK20 on IHC in Ta NMIBC.

**Supplementary Figure 4:** There was a significant difference in RNA expression of CK5 between patients that had 100% of CK5 IHC expression (combinations of CK5 IHC patterns: ‘normal’, ‘rising’, or ‘full-thickness’) and patients that had <50% presence of CK5 IHC expression (unpaired t test: p=0.02*). Data were visualized using the 2–ΔΔCt method for relative quantifications compared to internal control with GAPDH and S18.

**Supplementary Figure 5:** Kaplan-Meier plot of recurrence-free survival (RFS) of all patients by clusters identified using hierarchical clustering: CK5 absent/CK20+, CK5 normal, CK5 normal/CK20+, CK5 normal/rising , and CK5 rising/full-thickness.: CK5 absent/CK20+, CK5 normal, CK5 normal/CK20+, CK5 normal/rising , and CK5 rising/full-thickness.
